# Effect of Hypoxia on Siglec-7 and Siglec-9 Receptors and Sialoglycan Ligands and Impact of Their Targeting on NK Cell Cytotoxicity

**DOI:** 10.3390/ph17111443

**Published:** 2024-10-28

**Authors:** Husam Nawafleh, Nagwa Zeinelabdin, Michelle K. Greene, Anitha Krishnan, Linus Ho, Mohamed Genead, Derek Kunimoto, Christopher J. Scott, Michael Tolentino, Salem Chouaib

**Affiliations:** 1 Thumbay Research Institute for Precision Medicine, Gulf Medical University, Ajman 4184, United Arab Emirates; husam@gmu.ac.ae (H.N.); nagwa@gmu.ac.ae (N.Z.); 2 Aviceda Glycotech, 97 Lisburn Road, Belfast BT9 7AE, UK; mgreene@avilectbio.com (M.K.G.); c.scott@qub.ac.uk (C.J.S.); 3 Aviceda Therapeutics Inc., Cambridge, MA 02142, USA; akrishnan@avicedarx.com (A.K.); lho@avilectbio.com (L.H.); mgenead@avicedarx.com (M.G.); dkunimoto@avicedarx.com (D.K.); mtolentino@avicedarx.com (M.T.); 4 Patrick G Johnston Centre for Cancer Research, Queen’s University Belfast, Belfast BT7 1NN, UK

**Keywords:** siglec receptors, sialoglycan ligands, hypoxia, NK cells, nanoparticles, immune synapses

## Abstract

Background/Objectives: Tumor microenvironmental hypoxia is an established hallmark of solid tumors. It significantly contributes to tumor aggressiveness and therapy resistance and has been reported to affect the balance of activating/inhibitory surface receptors’ expression and activity on NK cells. In the current study, we investigated the impact of hypoxia on the surface expression of Siglec-7 and Siglec-9 (Sig-7/9) and their ligands in NK cells and tumor target cells. The functional consequence of Siglec blockage using nanoparticles specifically designed to target and block Sig-7/9 receptors on NK cell cytotoxicity was elucidated. Methods: CD56⁺ CD3^−^ NK cells were isolated from PBMCs along with an NK-92 clone and used as effector cells, while MCF-7 and K562 served as target cells. All cells were incubated under normoxic or hypoxic conditions for 24 h. To assess Siglec-7 and Siglec-9 receptor expression, U937, NK-92, and primary NK cells were stained with PE-labeled antibodies against CD328 Siglec-7/9. Interactions between Siglec-7/9 and their sialylated ligands, along with their functional impact on NK cell activity, were evaluated using polymeric nanoparticles coated with a sialic acid mimetic. Immunological synapse formation and live-cell imaging were performed with a ZEISS LSM 800 with Airyscan at 10× magnification for 24 h. Results: Our data indicate that hypoxia had no effect on the expression of Siglec-7/9 receptors by NK cells. In contrast, hypoxic stress resulted in an increase in Siglec-7 sialoglycan ligand expression by a sub-population of NK target cells. Using polymeric nanoparticles coated with a sialic acid mimetic that binds both Siglec-7 and -9 (Sig-7/9 NP), we demonstrated that incubation of these nanoparticles with NK cells resulted in increased immunological synapse formation, granzyme B accumulation, and killing of NK target cells. These studies indicate that hypoxic stress may have an impact on NK cell-based therapies and highlight the need to consider the hypoxic microenvironment for tumor-specific glycosylation. Conclusions: Our findings point to the role of Siglec–sialylated glycan interactions in hypoxic stress-induced NK cell dysfunction and recommend the potential integration of the manipulation of this axis through the targeting of Siglecs in future cancer immunotherapy strategies.

## 1. Introduction

Although new immunotherapy approaches have improved the survival of many patients with advanced malignancies, the high prevalence of non-responders also provides a strong reminder that we possess only a partial understanding of the events underlying the adaptive and innate immune resistance of tumors. In fact, several mechanisms are associated with the acquisition of tumor cell immune evasion and resistance [1,2,3]. Natural killer (NK) cells are lymphoid cells that are considered to be major innate effector cells, promoting antitumor activity. The lytic functions of these cells are regulated by a balance of activating and inhibiting signals originating from membrane receptors [4]. Despite their antitumor role, their contribution to inhibiting solid tumor progression can be abrogated. Indeed, the immunosuppressive tumor microenvironment (TME) is involved in tumor evasion from NK cell-mediated killing through several cellular and metabolic mechanisms. Emerging evidence has now emphasized how TME hypoxia modulates tumor cells to escape immune surveillance and, in particular, cell-mediated cytotoxicity [4,5]. TME hypoxia is a prominent feature of solid tumors and is involved in fostering the neoplastic process and in the modulation of immune reactivity. It correlates with increased risk of invasion, metastasis, tumor progression, treatment failure, and patient mortality [6]. This is, in part, due to the inappropriate local immune reaction and resistance of hypoxic tumor cells. Hypoxic stress, through its ability to induce tumor resistance and to regulate the differentiation and function of immune-suppressive cells, plays a prominent role in shaping the NK cell phenotype and function. We and others have shown that hypoxic tumors secrete cytokines and growth factors capable of creating a pro-metastatic milieu and reducing the cytotoxic function of NK cells [4,7,8,9]. Sialic acid-binding immunoglobulin-like lectins (Siglecs) 7 and 9 are immunoregulatory receptors that are found on a range of immune cells, including NK cells [10]. Siglecs 7 and 9 negatively regulate the function of NK cells and modulate the immune response through direct interaction with cell-surface sialic acid ligands, which are frequently upregulated in the TME. A recent study demonstrated that tumor cells upregulate sialylated glycans, which counteract NK cell-induced killing via the Siglec–sialylated glycan interaction [11,12]. Targeting Siglec-7 and Siglec-9 has therefore emerged as a novel therapeutic approach to enhance the immune response of NK cells to cancer. This distinct glycan-mediated pathway of immune suppression is less studied compared to other inhibitory mechanisms, making Siglec receptors a unique and important target for further investigation, particularly in hypoxic TME. In this current work, we studied the impact of hypoxia on the surface expression of Siglec-7/9 and their ligands in immune cells and tumor cells. To modulate the cytotoxic activity of NK cells, we developed a Siglec-7/9-targeting nanoparticle to display sialic acid mimetic ligands. Our findings point to the role of Siglec–sialylated glycan interactions in hypoxic stress-induced NK cell dysfunction and recommend the potential integration of the manipulation of this axis through the targeting of Siglecs in future cancer immunotherapy strategies.

## 2. Results

### 2.1. Hypoxia Does Not Change the Expression Levels of Siglec-7 and Siglec-9 Receptors on Immune Cells

**Figure 1:**
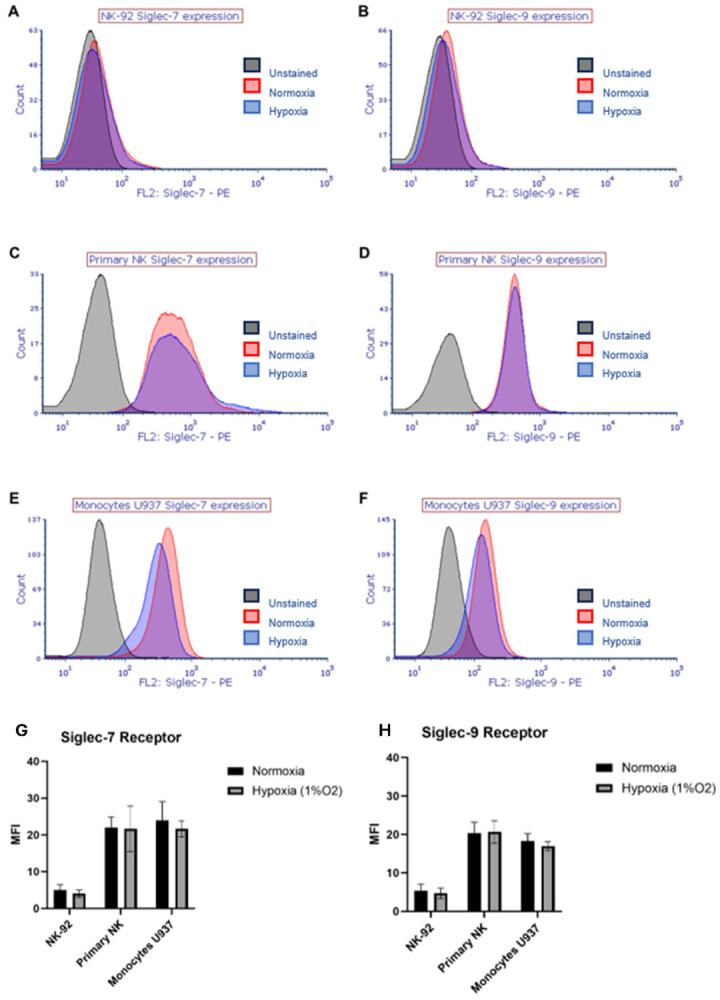
The effect of hypoxia on the surface expression of Siglec-7 and Siglec-9 receptors on immune cells. Histograms of one representative experiment show the effect of hypoxia (1% oxygen) for 24 h in comparison with normoxia (21% oxygen) on the surface expression of Siglec-7 and Siglec-9. (**A**,**B**) NK-92; (**C**,**D**) primary NKs; (**E**,**F**) U937 monocytes cell line. The bar charts represent the mean of the Median Fluorescent Intensity (MFI) of Siglec-7 Receptor (**G**) and Siglec-9 Receptor (**H**) on the surface of the indicated effector cells. U937 monocytes were used as a positive control while unstained samples for each experiment were used as negative controls. Error bars represent SEM of three independent experiments.

To investigate the impact of hypoxia on Siglec-7 and Siglec-9 expression in immune cells, we analyzed the levels of these receptors in NK-92 cells, and primary NK cells from healthy donors under both normoxic and hypoxic conditions. It is known that the monocytes express high levels of Sig-7/9; therefore, U937 monocyte cell line was used as a positive control [13]. Previous studies have indicated that NK-92 cells lack detectable levels of Siglec-7 and Siglec-9 under normoxic conditions, unlike primary NK cells. Nevertheless, their expression in hypoxic conditions has not been investigated [14]. Consistent with these findings, flow cytometry analysis confirmed the absence of Siglec-7 and Siglec-9 on NK-92 cells, while primary NK cells expressed both receptors (Figure 1A–H). Furthermore, hypoxia did not induce an increase in Siglec-7 or Siglec-9 expression on NK-92 cells (Figure 1A,B), nor did it alter the expression levels of these receptors on primary NK cells (Figure 1C,D). Examination of U937 cells revealed expression of Siglec-9 and Siglec-7 under normoxic conditions (Figure 1E,F), and hypoxia did not affect their expression levels.

### 2.2. Hypoxia Increases the Total Expression of Siglec-7 Ligand on the K562 Cell Line but Not on the MCF-7 Cell Line

**Figure 2:**
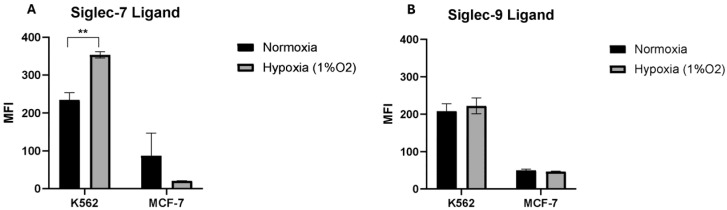
The effect of hypoxia on the surface expression of Siglec-7 and Siglec-9 ligands on tumor cell lines. The bar charts represent the Median Fluorescent Intensity (MFI) of (**A**) Siglec-7 ligand and (**B**) Siglec-9 ligand on the surface of K562 and MCF-7 cell lines in hypoxia and normoxia. Error bars represent SEM of three independent experiments. (**) *p* value ≤ 0.01 based on unpaired *t*-test.

We examined the surface expression of Siglec-7 and Siglec-9 ligands on K562 and MCF-7 cells using fluorescently labeled human Siglec-7 and 9-Fc chimera proteins. In line with what was previously shown, K562 expresses high Siglec-7 and Siglec-9 ligands, while MCF-7 expresses low Siglec-7 and Siglec-9 ligands [15]. Under hypoxic conditions, we observed a significant increase in Siglec-7 ligand expression on K562 cells, while Siglec-9 ligand expression remained unchanged (Figure 2). In contrast, no significant differences in the expression of either ligand were observed in MCF-7 cells.

### 2.3. Hypoxia Promotes High Siglec-7 Ligand Expression on a Sub-Population of K562 Cells

**Figure 3:**
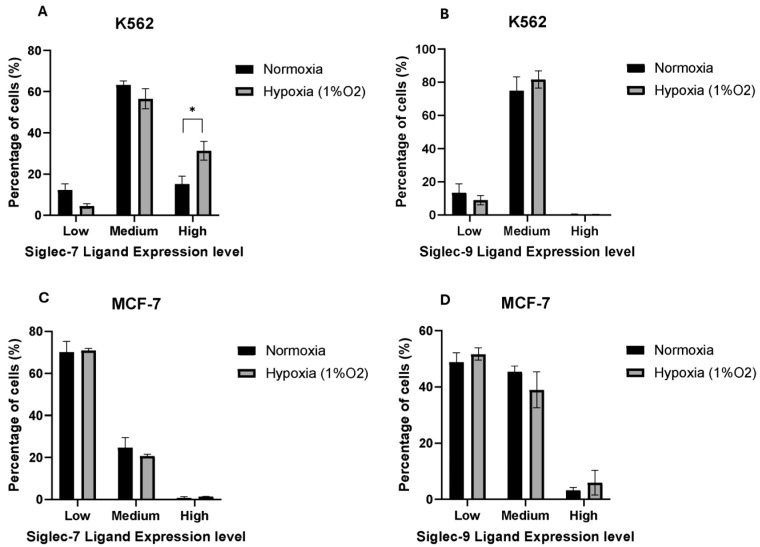
The effect of hypoxia on the regulation of Siglec-7 and Siglec-9 ligands on the surface of the K562 and MCF-7 cell lines. The bar charts show the percentages of K562 cells that express Siglec-7 ligand (**A**) and Siglec-9 ligand (**B**), as well as MCF-7 cells that express Siglec-7 ligand (**C**) and Siglec-9 ligand (**D**) at different levels: low, medium, and high expression levels, as assessed by flow cytometry. Error bars represent SEM of three independent experiments. (*) *p* value ≤ 0.05 based on unpaired *t*-test.

We investigated whether variable expression levels of Siglec-7 or Siglec-9 ligands could be detected in different cell lines. Under hypoxic conditions, we observed an increase in a sub-population of K562 cells showing higher expression of Siglec-7 ligand (Figure 3A and Figure S1A). However, this enhanced expression was not evident for Siglec-9 ligand in K562 cells (Figure 3B). Interestingly, hypoxia did not influence the surface expression levels of Siglec-7 or Siglec-9 ligands on MCF-7 cells (Figure 3C,D and Figure S1B). These findings suggest that hypoxia selectively promotes high Siglec-7 ligand expression in K562 cells, highlighting potential implications for understanding and targeting Siglec signaling pathways in cancer therapeutics.

### 2.4. Siglec-7/9-Blocking Nanoparticles Increase the Immune Reactivity of Primary NK Cells

**Figure 4:**
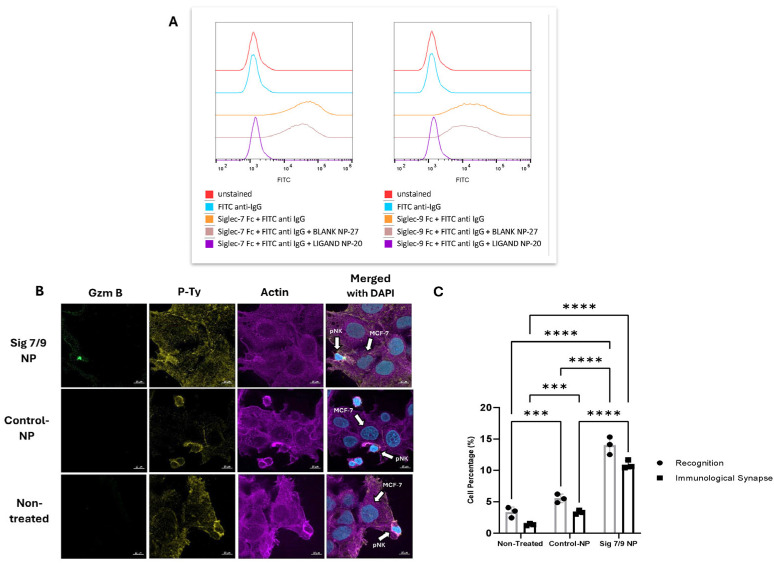
Immunological synapse formation. (**A**) Inhibition of Siglec-7/Fc chimera and Siglec-9/Fc chimera binding to K562 cells by Sig-7/9 NPs. Representative histogram shown from three independent experiments. (**B**) Confocal imaging of the accumulation of granzyme B, actin, and phospho-tyrosine between Sig-7/9 NPs and Control NPs treated and non-treated peripheral NK and MCF-7 cells representing immunological synapse formation and/or recognition. Scale bar = 10 µm. (**C**) Quantification of the treated and non-treated peripheral NK cells recognizing and/or forming an immunological synapse with MCF-7. Error bars represent SEM of three independent experiments. Statistical significance was determined using two-way ANOVA. (***) *p* value ≤ 0.001; (****) *p* ≤ 0.0001.

**Figure 5:**
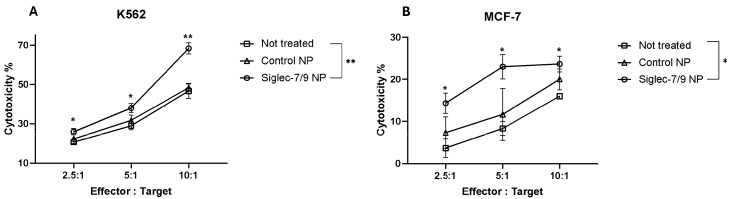
The effect of nanoparticles on primary NK-mediated cytotoxicity. Target tumor cell lines (**A**) K562 and (**B**) MCF-7 were co-cultured with treated primary NK cells and assessed using LDH cytotoxicity assay. Error bars represent SEM of three independent experiments. Statistical significance was determined using two-way ANOVA. (*) *p* value ≤ 0.05; (**) *p* value ≤ 0.01.

**Figure 6:**
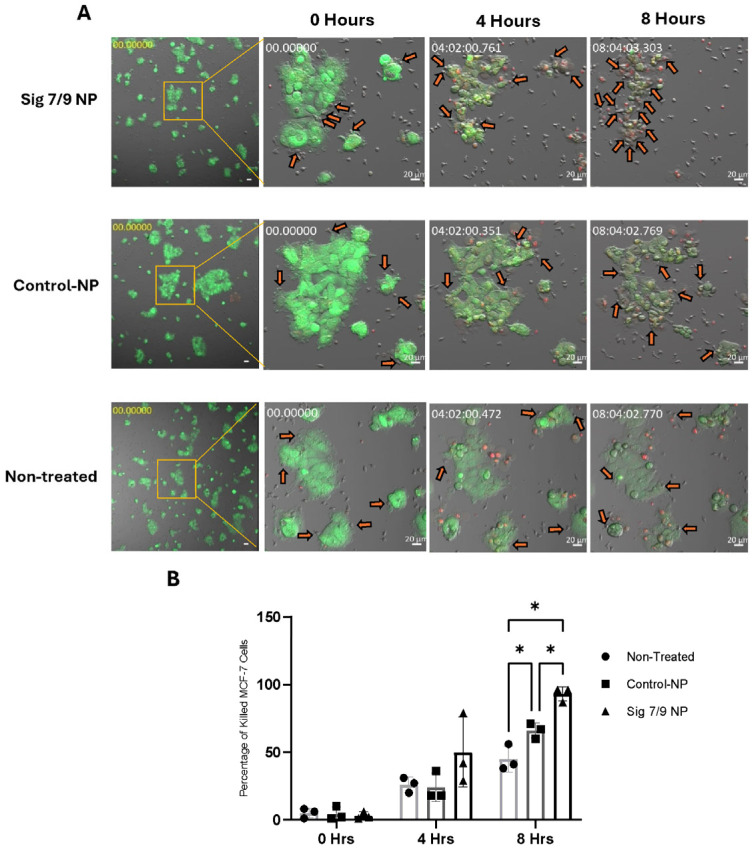
Live-cell imaging of peripheral NK cytotoxicity treated with Sig-7/9 NPs, Control NPs and non-treated peripheral NK cytotoxicity on MCF-7 cells. MCF-7 cells labeled with CMFDA green co-cultured with primary NK labeled with CMTMR orange. Dead cells shown in red. (**A**) Representative confocal images taken at different time points (0, 4 and 8 h). Scale bar = 100 µm. Orange arrows are pointing at the pNK cells forming immunological synapses with the target cells. (**B**) The percentages of killed MCF-7 cells by the treated and non-treated peripheral NK. Error bars represent SEM of three independent experiments. Statistical significance was determined using two-way ANOVA. (*) *p* value ≤ 0.05.

Interactions between Siglec-7 and Siglec-9 with their sialylated ligands are known to inhibit the cytotoxicity of immune cells. We next investigated whether a blockade of Siglec-7 and Siglec-9 receptors on primary NK cells could enhance their cytotoxic phenotype. To achieve this, we developed polymeric nanoparticles coated with a sialic acid mimetic capable of binding to Siglec-7 and Siglec-9. We first confirmed the ability of these nanoparticles to block the interaction between recombinant soluble Siglec proteins (as surrogates for NK cell-expressed Siglecs) and sialic acids on tumor cells. Compared to control nanoparticles lacking the inhibitory ligand (Control NP), the sialylated nanoparticles (Sig-7/9 NP) significantly reduced the binding of Siglec-7 and Siglec-9 Fc chimera proteins to K562 cells (Figure 4A), indicating effective Siglec occupancy. Next, we evaluated whether Sig-7/9 NP could similarly block endogenous Siglec receptors on the surface of primary NK cells and thus leads to augmenting its immune reactivity. Preincubation of primary NK cells with Siglec-7/9 nanoparticles (NPs) for 16 h enhanced both target recognition and immunological synapse formation. The immunological synapse was marked by the accumulation of actin, phosphotyrosine, and granzyme B at the interface between NK and MCF-7 cells. Notably, NK cells can recognize target cells without releasing granzyme B if cytotoxicity is suppressed by inhibitory receptors like Siglecs. Our results demonstrate that blocking Siglec receptors with nanoparticles not only enhanced recognition but also promoted effective synapse formation, suggesting that disrupting Siglec signaling restores NK cell cytotoxic function against target cells (Figure 4B,C). Furthermore, the enhanced accumulation of granzyme B, actin, and phospho-tyrosine between primary NK cells and MCF-7 cells suggests not only recognition but also immunological synapse formation in Sig-7/9 NP-treated cells compared to Control NPs and non-treated cells (Figure 4B,C). To assess the impact of Sig-7/9 NPs on primary NK cell cytotoxicity against target cells, LDH cytotoxicity assays were performed at various effector to target (E:T) ratios (2.5:1, 5:1, and 10:1). Sig-7/9 NP-treated primary NK cells exhibited significantly increased cytotoxicity against K562 and MCF-7 target cells compared to Control NPs and non-treated cells (Figure 5A,B). Live-cell imaging analysis at an E:T ratio of 10:1 further confirmed enhanced cytotoxicity of Sig-7/9 NP-treated NK cells, with a notable increase in killing efficiency against MCF-7 cells over an 8 h period (Figure 6A,B).

## 3. Discussion

New therapies that promote antitumor immunity (for example, antibodies against Programmed Death 1, or PD-1) have been recently developed. Although these therapies have led to some unprecedented successes, only a minority of patients with cancer benefit from such treatments, highlighting the need to identify new cells and molecules that can be exploited in the next generation of cancer immunotherapy [16]. Recent studies highlighting the role of inhibitory Siglecs in immune evasion in cancer suggest that Siglec may represent a new pathway for immune checkpoint inhibition [17].

Hypoxia is known to be associated with tumor progression and poor clinical outcomes [18]. It induces a number of events in the TME that lead to the selection and expansion of aggressive clones from heterogeneous tumor cells and promote a lethal phenotype. In previous studies, we have demonstrated that hypoxic stress was associated with an impairment of cell-mediated lysis through different mechanisms involving, in particular, the induction of PD-L1 [19]. More importantly, we showed that hypoxia-induced autophagic degradation of granzyme B in tumor target cells is a new mechanism of hypoxic tumor cell escape from NK cell-mediated lysis [20]. In addition, we provided the first evidence that the hypoxic microenvironment negatively affects the immune surveillance of tumors by NK cells through the modulation of Cx43-mediated intercellular communications [21].

It is increasingly evident that tumor cells have evolved to exploit hyper-sialylation as a potent form of immunosuppression, tricking the immune system into recognizing them. Cancer-specific changes in sialylation are recognized by cell membrane-bound immune checkpoint receptors and their soluble proteins, inhibiting immune activation and actively contributing to cancer progression and immunity. A better understanding of the different functions of Siglecs based on their expression in various cell types and the mechanisms by which they promote tumor immune escape to reduce the adverse reactions of tumor drugs is currently of major interest. Previous studies have highlighted variability in ligand expression among different cancer cell types under normoxic conditions [15,22].

Our findings demonstrate that hypoxia does not modulate the expression of Siglec-7 and Siglec-9 receptors on immune cells under the experimental conditions employed. This underscores the stability of Siglec receptor expression in immune cells despite changes in oxygen levels, which is critical for understanding their role in immune responses and potential therapeutic interventions targeting the Siglec/sialic acid axis in cancer. However, we found that hypoxia can influence malignant cells by augmenting the expression and presentation of Siglec ligands, thereby allowing them to evade NK cell-mediated lysis [23]. Hypoxia has been shown to affect the structure and presentation of sialoglycans on tumor cells, resulting in enhanced overall cell surface sialylation [24], likely due to the effects on the expression of sialyl-transferases [25,26].

Moreover, our results indicate that hypoxia specifically upregulates Siglec-7 ligand expression on K562 cells. While hypoxic stress does not impact the expression of Siglec-7 and Siglec-9 receptors by NK cells, it does increase Siglec-7 ligand expression in a sub-population of K562 cells. These findings highlight the potential role of hypoxia in modulating Siglec-mediated interactions between immune cells and cancer cells, emphasizing the implications for immune evasion mechanisms and therapeutic strategies targeting the Siglec/sialic acid axis in cancer.

More importantly, using polymeric nanoparticles coated with a sialic acid mimetic that binds Siglec-7 and -9, we demonstrated that these nanoparticles, when incubated with NK cells, increased immunological NK cell synapse formation, granzyme B accumulation, and promoted target cell killing, even in MCF-7 target cells with low Siglec-7/9 ligand expression. This further confirms that, in addition to the regulation of tumor cell phenotype, hyper-sialylation influences signaling pathways in the context of immunological synapses and thereby potentiates tumor immune evasion. The Sig-7/9 NP markedly reduces the binding of Siglec-7 and -9 proteins to MCF-7 cells, indicating that Sig-7/9 NP are capable of interfering with the Siglec–sialic acid axis. This is consistent with the fact that tumor cells may escape from innate immune attack and can also be associated with aberrant expression of sialoglycan ligands that engage the MHC class I-independent Siglec-7 and -9 receptors to govern NK cell inhibition. It has been reported that the structure and signaling motifs of Siglec receptors display great resemblance to PD-1 [27]. Therefore, the immune inhibitory Siglecs are currently envisioned as potential immune checkpoint receptors that can be targeted in cancer [28,29,30]. It would be of major interest to elucidate the molecular and cellular basis of TME-induced regulation of Siglec expression and signaling in the TME. The Siglec-mediated modulation of NK cell function needs to be further explored in the context of TME complexity and heterogeneity to evaluate the potential of targeting this pathway in patients.

Our data clearly indicate that Sig-7/9 NP-treated NK cells show a significant increase in the synapse formation with MCF-7 targets. This is in agreement with the report of Jandus et al. (2014) where immunodeficient (NSG) mice reconstituted with human hematopoietic stem cells and inoculated with human tumor cells demonstrated a sialoglycan-dependent inhibition of NK cell-mediated tumor cell killing [15]. In another study by Bertozzi and colleagues, artificially increasing the density of sialoglycan-SAMPs (self-associated molecular patterns) on the surface of cancer cells lead to Siglec-7 engagement on NK cells and the subsequent inhibition of both their antibody-dependent and -independent cytotoxic activities [31]. Our findings demonstrate that Siglec-7/9-blocking nanoparticles effectively enhance the immune reactivity of primary NK cells against tumor target cells, highlighting their potential as therapeutic agents to boost NK cell-mediated cytotoxicity in cancer immunotherapy.

Multiple clinical trials have tried to stimulate the immune system with NK cell therapy since NK-cell-mediated cytotoxicity is essential for the elimination of cancer cells. Human NK cells constitutively express inhibitory Siglec-7 and Siglec-9 [32]. In addition, it has been found that in cancer, Siglec-9 is upregulated on peripheral NK cells, mainly on CD56dim CD16+ NK cells [15]. The in vitro cytotoxicity of these NK cells against tumor cells (K562) was increased when Siglec-7 or Siglec-9 were blocked by Fab fragments [15]. Several studies strongly suggested that the Siglec–sialic acid axis can act as a potential target for cancer immunotherapy. Therefore, targeting Siglec-7 and Siglec-9 has emerged as a novel therapeutic approach to enhance the immune response of NK cells to cancer. Indeed, the PD1 receptor and Siglec-7/9 have a homologous ITIM-ITSM cytosolic sequence motif and are expressed on a variety of immune cells. As Siglec-7/9 are immune modulatory receptors, it is possible that these Siglecs function as checkpoint receptors that contribute to cancer immune evasion, analogous to PD-1.

The combination of strategies to potentiate the effectiveness of NK cells in the TME offer highly attractive therapeutic approaches to enhance immune responses in tumors. As well as using Siglec-7/9 abrogation molecules such as the nanoparticles deployed here, there is much interest currently in the development of chimeric antigen receptor (CAR)-NK cells as a promising next generation of cellular therapy. Preclinical data on the use of these cells have demonstrated efficacy for a variety of cancers and target antigens. In this regard, the novel CAR-NK cell-based strategies should also aim to tip the balance between activating and inhibitory receptors signals. In line with our findings, we speculate that developing innovative strategies regulating the NK decision to kill by targeting Siglec-7 and Siglec-9 using modified nanoparticles could be highly attractive—particularly as they can have broader specificity than antibodies targeting either one or other TME-associated Siglecs.

## 4. Materials and Methods

### 4.1. Cell Lines and Culture Conditions

The human breast cancer-derived cell line MCF-7, the monocytic lymphoma cell line U937, the chronic myeloid leukemia cell line K562, and the immortalized natural killer cell line NK-92 were gifted from INSERM, Gustave Roussy, France. All cell lines were grown in RPMI 1640 GlutaMax Medium (Cat. No. 61870010, Gibco, ThermoFisher Scientific, Waltham, MA, USA) supplemented with 10% fetal bovine serum (FBS) (Cat. No. 10270106, Gibco), 1% sodium pyruvate (Cat. No 11360039, Gibco), and 1% penicillin–streptomycin (Cat. No. 15140122, Gibco) and cultured at 37 °C in a humidified incubator (ESCO Cell Culture incubator, Marietta, OH, USA) at 5% CO_2_ and 21% O_2_.

### 4.2. Isolation of Primary Natural Killer Cells

Blood from healthy subjects was collected following informed consent. Peripheral blood was collected in heparin lithium tubes, and peripheral blood mononuclear cells (PBMCs) were obtained by density centrifugation using Histopaque^®^-1077 (Cat. No. 10771-100ML, Sigma, Livonia, MI, USA). CD56^+^ CD3^−^ NK cells were isolated from the PBMCs by negative selection using the MojoSort™ Human NK Cell Isolation Kit (Cat. No. 480053, BioLegend, San Diego, CA, USA) according to the manufacturer’s instructions. The purity of the isolated cells was >95% as assessed by AF700-conjugated CD56 and FITC-conjugated CD3 co-staining using flow cytometry (Cat. No. 51997S and 86774S, Cell Signaling Technology, Danvers, MA, USA) (Supplementary Figure S2).

### 4.3. Hypoxia Incubation Conditions

Cells were seeded overnight in normoxia, then incubated under hypoxic conditions in the humidified Whitley-H35 Hypoxystation (Don Whitley Scientific Limited, West Yorkshire, UK) at 37 °C, 5% CO_2_, and 1.0% O_2_ for 24 h before collection.

### 4.4. Assessment of Siglec-7 and Siglec-9 Receptors on Immune Cells in Hypoxia

Hypoxic and normoxic U937, NK-92, and primary NK cells were individually stained with PE anti-human CD328 (Siglec-7) Antibody (Cat. No. 339204, BioLegend, San Diego, CA, USA) or PE anti-human Siglec-9 Antibody (Cat. No. 351504, BioLegend, San Diego, CA, USA). The isotype control used was PE-conjugated IgG1 MOPC-21 (Cat. No 400112, BioLegend, San Diego, CA, USA). The cells were stained for 1 h at 4 °C. The nonviable cells and the double cells were excluded from the analysis and the intensity of the expression was then analyzed.

### 4.5. Assessment of Siglec-7 and Siglec-9 Ligands on Tumor Cells in Hypoxia

Recombinant human Siglec-7/Fc chimera (Cat. No. 1138-SL, R&D Systems, Minneapolis, MN, USA) or Siglec-9/Fc chimera (Cat. No. 1139-SL, R&D Systems, Minneapolis, MN, USA) was complexed with PE-conjugated anti-human IgG (Cat. No. FAB 110P, R&D Systems, Minneapolis, MN, USA) for 1 h at 4 °C. The hypoxic and normoxic cells were stained with the antibody complex for 1 h at 4 °C. The adherent cell line MCF-7 was detached from the culture plate using VerseneTM (Cat. No. 15040066, ThermoFisher Scientific, Waltham, MA, USA). Fluorescent staining was assessed using an S3e cell sorter (BioRad, Hercules, CA, USA), and the data were analyzed using FCS Express 7–Research edition. Nonviable cells and double cells were excluded from analysis, and the intensity of expression was then analyzed.

### 4.6. Assessment of Siglec Fc Chimera Binding to K562 Cells +/− Nanoparticles

Recombinant human Siglec-7/Fc chimera or Siglec-9/Fc chimera (5 mL of 50 mg/mL stock) were complexed for 15 min at 4 °C with FITC-conjugated anti-human IgG Fc antibody (5 mL of 100 mg/mL stock, Cat. No. 410720, Biolegend) in 90 mL of 0.5% BSA/PBS (FACS buffer). Control NPs or Sig-7/9 NPs (50 mL of 2.2 mg solids/mL stock) were then incubated with the above for 15 min at 4 °C, followed by addition of K562 cells (50 mL of 6 × 10^6^ cells/mL stock) for a further 45 min at 4 °C. Cells were washed × 2 in FACS buffer and FITC fluorescence was assessed on an Accuri C6 Plus flow cytometer (BD). A minimum of 10,000 events per sample was acquired after setting gates to exclude debris, dead cells (through staining with Zombie NIR™ fixable viability dye (Cat. No. 423105, Biolegend), and doublets. Data analysis was performed using FlowJo software (version 10.8.1).

### 4.7. Treatment of Natural Killer Cells with Nanoparticles

Peripheral NK cells were seeded into each of three wells of glass 24-well plates (Cat. No. 0030741021, Eppendorf, Germany). One well of seeded NK cells was left untreated, the second well was treated with ligand-conjugated nanoparticles (Sig-7/9 NPs), and the third well was treated with blank nanoparticles (Control NPs; Aviceda Therapeutics, Cambridge, MA, USA), all for 16 h at 1 mg/mL concentration of nanoparticles. Treated and untreated NK cells were then washed twice with 1× PBS. Siglec-7/9 NPs were prepared using a core of polyethylene glycol (PEG) and poly-lactic co-glycolic acid (PLGA) polymers to which high affinity ligands were covalently attached.

### 4.8. Immunological Synapse Formation

MCF-7 cells (5 × 10^4^) were seeded into each of three wells of glass 24-well plates (Cat. No. 0030741021, Eppendorf, Germany) and incubated overnight in RPMI media. Peripheral NK cells (5 × 10^5^ cells/well) treated or not with nanoparticles were co-cultured with the MCF-7 cells for 30 min and then fixed with 4% PFA for 10 min. Cells were permeabilized with 0.1% Triton-X for 15 min followed by blocking with 2% BSA for one hour. Cells were stained with the following primary and secondary antibodies: rabbit anti-granzyme B 1:200 (Cat. No. 17125 Cell Signaling, Danvers, MA, USA), mouse anti-phospho-tyrosine 1:1600 (Cat. No MA1-10443 ThermoFisher Scientific, Waltham, MA, USA), Phalloidin Alexa-Flor-647 1:20 (Cat. No. 8940 Cell Signaling, Danvers, MA, USA) and DAPI (Cat. No. D1306 ThermoFisher Scientific, Waltham, MA, USA), goat anti-rabbit Alexa Fluor-488 1:1000 (Cat. No. A11034 ThermoFisher Scientific, Waltham, MA, USA), and goat anti-mouse Alexa Fluor 568 1:1000 (Cat. No. A11004 ThermoFisher Scientific, Waltham, MA, USA). Confocal images were captured using ZEISS LSM 800 with Airyscan at 63× objective to analyze immunological synapse formation and target recognition. Immunological synapses were identified as cells showing the accumulation of actin, phosphor-tyrosine, and granzyme B at the interface between NK and target cells. In contrast, target recognition was defined as the accumulation of actin and phosphotyrosine at the interface without granzyme B, indicating recognition without the initiation of cytotoxic activity.

### 4.9. NK Cell-Mediated LDH Cytotoxicity Assay

Primary NK cell-mediated cytotoxicity was assessed using the CyQUANT™ LDH Cytotoxicity Assay Kit (Cat. No. C20301, Invitrogen, ThermoFisher Scientific, Waltham, MA, USA). The target cells MCF-7 and K562 were seeded into round-bottom 96-well plates at a density of 2 × 10^3^ cells per well and allowed to adapt for 24 h. Following treatment with ligand-conjugated nanoparticles or blank nanoparticles, the NK cells were co-cultured with target cells at different effector to target ratios (E: T; 2.5:1, 5:1, and 10:1) for 4 h and the LDH activity in cell supernatants was measured. The cytotoxicity of the NK cells against K562 or MCF-7 cells were calculated as per the kit protocol.

### 4.10. Live-Cell Imaging

MCF-7 cells (5 × 10^4^) were seeded in 24-well cell imaging glass-bottom plates (Cat. No. 0030741021, Eppendorf, Germany) and incubated overnight in RPMI media. Seeded MCF-7 cells were labeled with CellTracker CMFDA Green at 5 µM final concentration (Cat. No. C7025, ThermoFisher Scientific, Waltham, MA, USA). Peripheral NK cells (5 × 10^5^) treated or not with nanoparticles were labeled with CellTracker CMTMR Orange at 5 µM final concentration (Cat. No. C2927, ThermoFisher Scientific, Waltham, MA, USA) and co-cultured with labeled MCF-7 cells in the presence of 200 IU IL-2. TO-PRO-3 Iodide (Cat. No. T3605, ThermoFisher Scientific, Waltham, MA, USA) was added to the media (1 µM final concentration) to stain dead nuclei. Co-cultured cells were incubated at 37 °C and visualized using ZEISS LSM 800 with Airyscan at 10× magnification for 24 h. Dead target cells were quantified at different time points (0.4 and 8 h).

### 4.11. Statistical Analysis

Differences between groups were analyzed by applying the unpaired *t*-test or two-way ANOVA using GraphPad Prism 9 software (San Diego, CA, USA). Differences with *p* values ≤ 0.05 at confidence levels greater than 95% were considered statistically significant. The results are represented as mean ± SEM (standard error of mean) for three independent experiments.

## 5. Conclusions

Our findings reveal the key role of Siglec—sialylated glycan interactions in hypoxia-induced NK cell dysfunction, a mechanism that promotes immune evasion by cancer cells. Hypoxia enhances these interactions, suppressing NK cell activation and cytotoxicity. Targeting this axis through Siglec inhibitors or monoclonal antibodies offers a promising therapeutic strategy to restore NK cell function. Integrating such approaches with existing immunotherapies, like checkpoint inhibitors, could further improve antitumor responses by countering the immune-suppressive effects of the hypoxic tumor microenvironment.

## Data Availability

The original contributions presented in this study are included in the article/Supplementary Materials. Further inquiries can be directed to the corresponding author.

## Supplementary Materials

The following supporting information can be downloaded at: https://www.mdpi.com/article/10.3390/ph17111443/s1, Figure S1: Gating strategy for the identification of low, medium, and high Siglec-7 ligand-expressing cells. title; Figure S2: Gating strategy for the identification and purity assessment of primary NK cells isolated from healthy donor blood.
