# Supplementary material for: Effect of Hypoxia on Siglec-7 and Siglec-9 Receptors and Sialoglycan Ligands and Impact of Their Targeting on NK Cell Cytotoxicity
Source: Pharmaceuticals (Basel). 2024 Oct 28;17(11):1443. doi: 10.3390/ph17111443 (PMC11597189; doi:10.3390/ph17111443)
Supplement: Supplementary file 1 [file pharmaceuticals-17-01443-s001.zip › pharmaceuticals-3195668-supplementary.pdf]

S1A

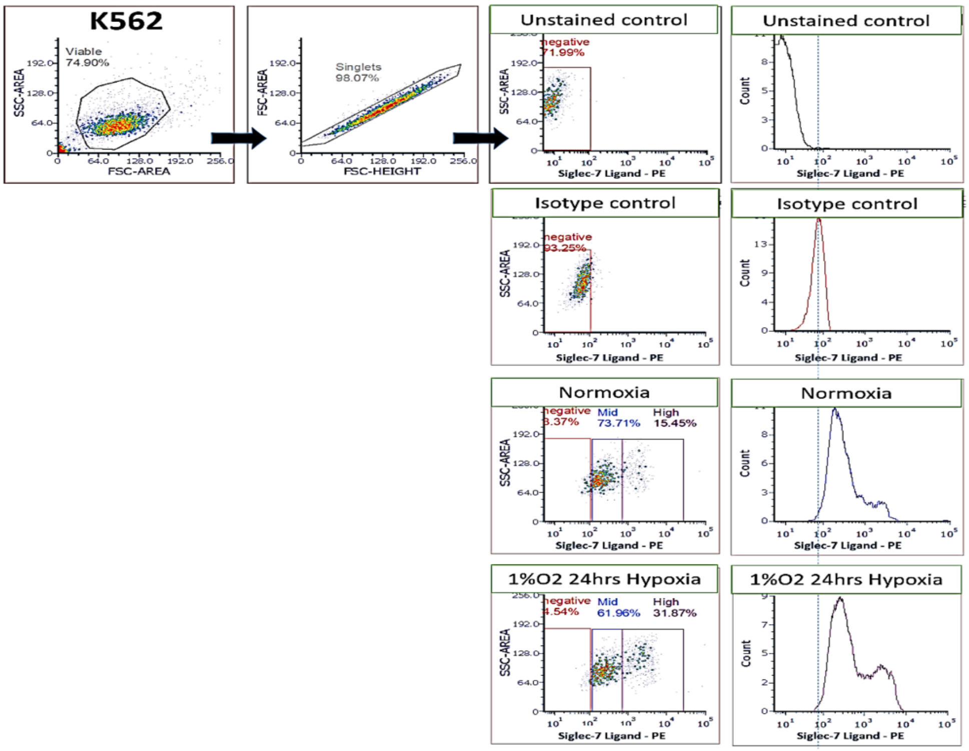

S1B

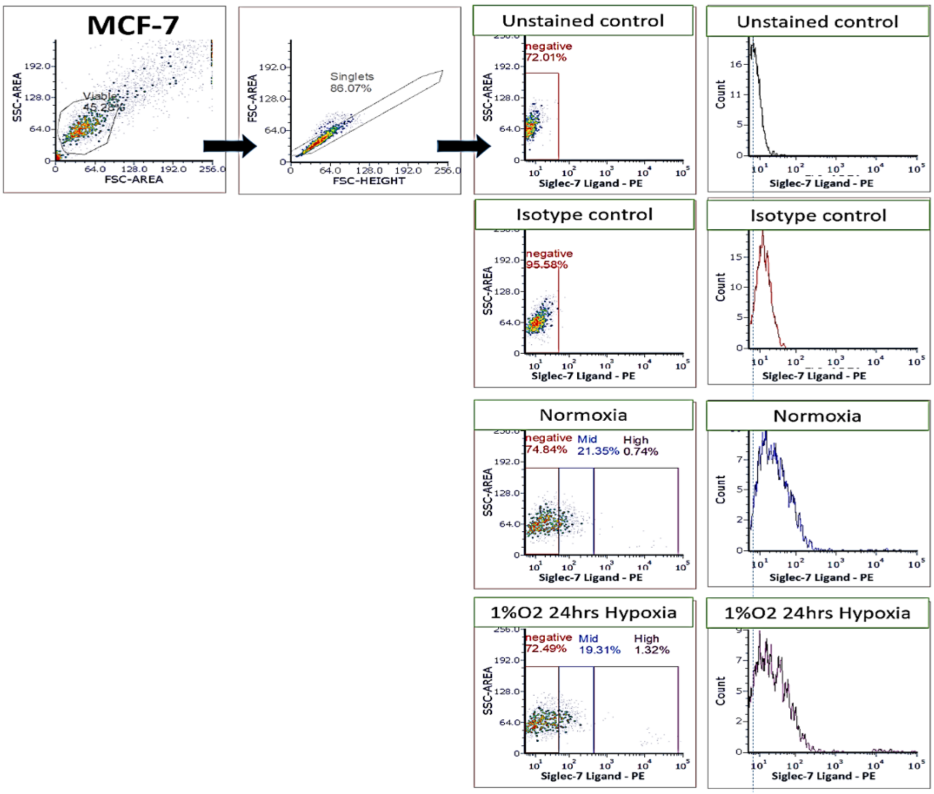

Figure S1. Gating strategy for the identification of low, medium, and high Siglec-7 ligand-expressing cells. The flow cytometry gating strategy used to classify K562 (S1A) and MCF-7 (S1B) cells based on their surface expression levels of Siglec ligands under normoxic and hypoxic conditions. This is one representative experiment showing how the cells were categorized into low, medium, and high expressing populations by fluorescence intensity. In K562 cells (S1A), hypoxia resulted in an increase in the proportion of high Siglec-7 ligand-expressing cells, while no significant changes were observed in MCF-7 cells (S1B). Gates were defined based on the matched isotype controls.

S2

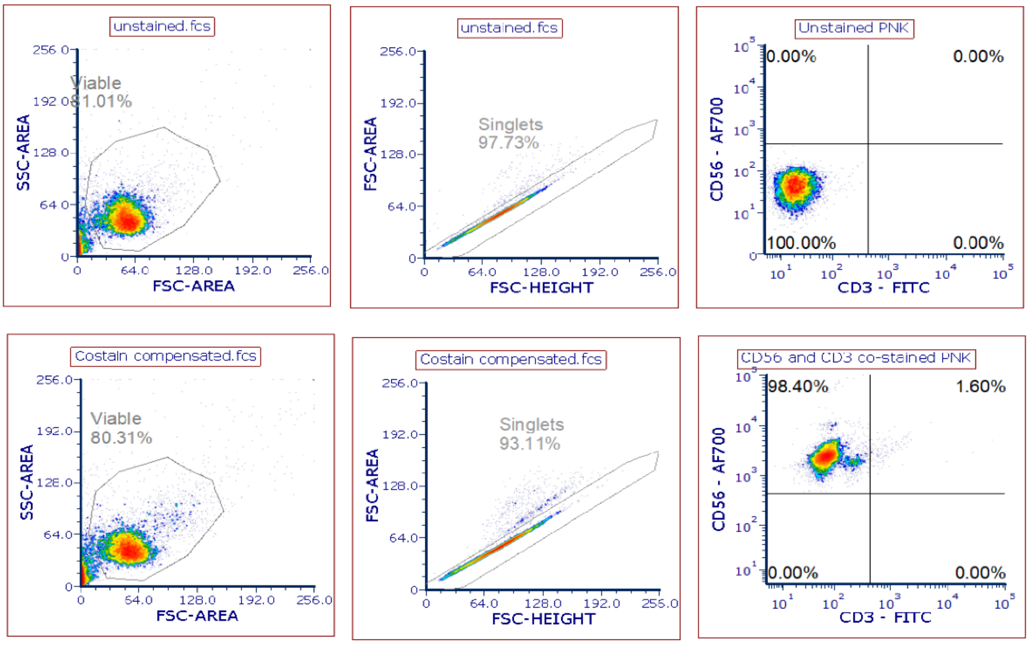

Figure S2. Gating strategy for the identification and purity assessment of primary NK cells isolated from healthy donor blood. The flow cytometry gating strategy used to identify primary NK cells based on the expression of CD56 and CD3. Cells were first gated on live, single cells, followed by gating on CD56+CD3− populations to identify NK cells. The purity of the NK cell isolation is shown, with one representative sample displaying 98.4% purity.
